# Efficient Charging of Li‐Ion Batteries with Pulsed Output Current of Triboelectric Nanogenerators

**DOI:** 10.1002/advs.201500255

**Published:** 2015-09-25

**Authors:** Xiong Pu, Mengmeng Liu, Linxuan Li, Chi Zhang, Yaokun Pang, Chunyan Jiang, Lihua Shao, Weiguo Hu, Zhong Lin Wang

**Affiliations:** ^1^ Beijing Institute of Nanoenergy and NanosystemsChinese Academy of ScienceBeijing100083P.R. China; ^2^ School of Materials Science and EngineeringGeorgia Institute of TechnologyAtlantaGA30332‐0245USA

**Keywords:** impedance match, Li‐ion battery, power utilization efficiency, pulsed current, triboelectric nanogenerator

## Abstract

The triboelectric nanogenerator (TENG) is a promising mechanical energy harvesting technology, but its pulsed output and the instability of input energy sources make associated energy‐storage devices necessary for real applications. In this work, feasible and efficient charging of Li‐ion batteries by a rotating TENG with pulsed output current is demonstrated. In‐depth discussions are made on how to maximize the power‐storage efficiency by achieving an impedance match between the TENG and a battery with appropriate design of transformers. With a transformer coil ratio of 36.7, ≈72.4% of the power generated by the TENG at 250 rpm can be stored in an LiFePO_4_–Li_4_Ti_5_O_12_ battery. Moreover, a 1 h charging of an LiCoO_2_–C battery by the TENG at 600 rpm delivers a discharge capacity of 130 mAh, capable of powering many smart electronics. Considering the readily scale‐up capability of the TENG, promising applications in personal electronics can be anticipated in the near future.

## 1 Introduction

Sustainable energy harvesting is becoming increasingly important because of the fast raising of portable electronics. For grid‐scale applications, efforts are being made to find alternatives, such as solar and wind, to traditional fossil fuel–based power systems; for electronic‐scale applications, the current trend is to harvest energy in various forms (thermal,^1^ mechanical,^2^ solar,^3^ etc.) from the working environment of the device so as to reduce or even replace the use of batteries, since which require frequent and inconvenient charging. Among various energy sources, mechanical energy, due to its universal availability, is of major importance, especially for personal electronics. Different mechanisms have been established for harvesting mechanical energy, including electromagnetic generator,^4^ electrostatic generator,^5^ piezoelectric nanogenerator,^6^ and the recently developed triboelectric nanogenerator (TENG).^7^ The TENG, based on the coupled effect of contact‐electrification and electrostatic induction, is especially promising, considering its demonstrated high power density, low‐cost, and environmental friendliness.^8^

However, several following challenges are required to be addressed. First, the energy harvested by the TENG from the environment is usually time‐dependent or climate‐dependent, and thus is not stable or even unpredictable. This also applies to solar, wind, and thermal energy sources. Therefore, energy storage by batteries or capacitors and associated power management circuits are required in order to obtain constant or controllable power supplies.[[qv: 7c]]^,^[[qv: 8b]] The subsequent challenge is to maximize the storing efficiency of the generated power. The TENG has an output characteristic of high voltage, large internal impedance, and small current.^9^ Optimized external loading impedance to maximize the power utilization of a TENG is around 10^5^–10^7^ Ω,[[qv: 9a]] much larger than that of batteries (10^−2^–10^2^ Ω). Therefore, even though the electrochemical cell itself can achieve energy efficiency higher than 90%, impedance match between the energy‐generating TENG and energy‐storing cell needs to be achieved so as to maximize the total efficiency.^10^ Finally, the TENG typically has a pulsed output. If, in certain cases, power management circuit is not applicable, the feasibility of charging batteries directly with pulsed current of a TENG needs to be demonstrated. The pulsed charging of batteries, such as lead‐acid and lithium‐ion batteries (LIBs), is one of the advanced charging techniques that can lower the concentration polarization of the cell, and thus improve the charging efficiency and battery lifetime.^11^

In this work, we demonstrated a feasible and efficient charging of LIBs with pulsed output current generated by a rotating TENG. Fast Li‐ion extractions of typical electrode materials, i.e., LiFePO_4_ and Li_4_Ti_5_O_12_, were achieved by the TENG at 250 rpm rotating speed. The estimated columbic efficiency of the TENG charging and the following 0.5 C discharging can be higher than 90%, comparable with that of constant current charging. More importantly, the improvement of power utilization efficiency (up to 72.4%) from the TENG to a home‐made LiFePO_4_–Li_4_Ti_5_O_12_ full cell was achieved by optimizing the coil ratio of a transformer. High efficiency was achieved when the impedance of the TENG is lowered close to that of battery cell. Subsequently, we showed that a 1 h charging of a commercial LIB by the TENG (600 rpm, 36.7 transformer coil ratio) can later deliver a discharge capacity about 130 mAh. Finally, general principles on maximizing the power utilization efficiency from TENG to batteries were discussed.

This is an open access article under the terms of the Creative Commons Attribution License, which permits use, distribution and reproduction in any medium, provided the original work is properly cited.

## 2 Results and Discussions

### 2.1 Output Characteristics of the TENG

**Figure 1:**
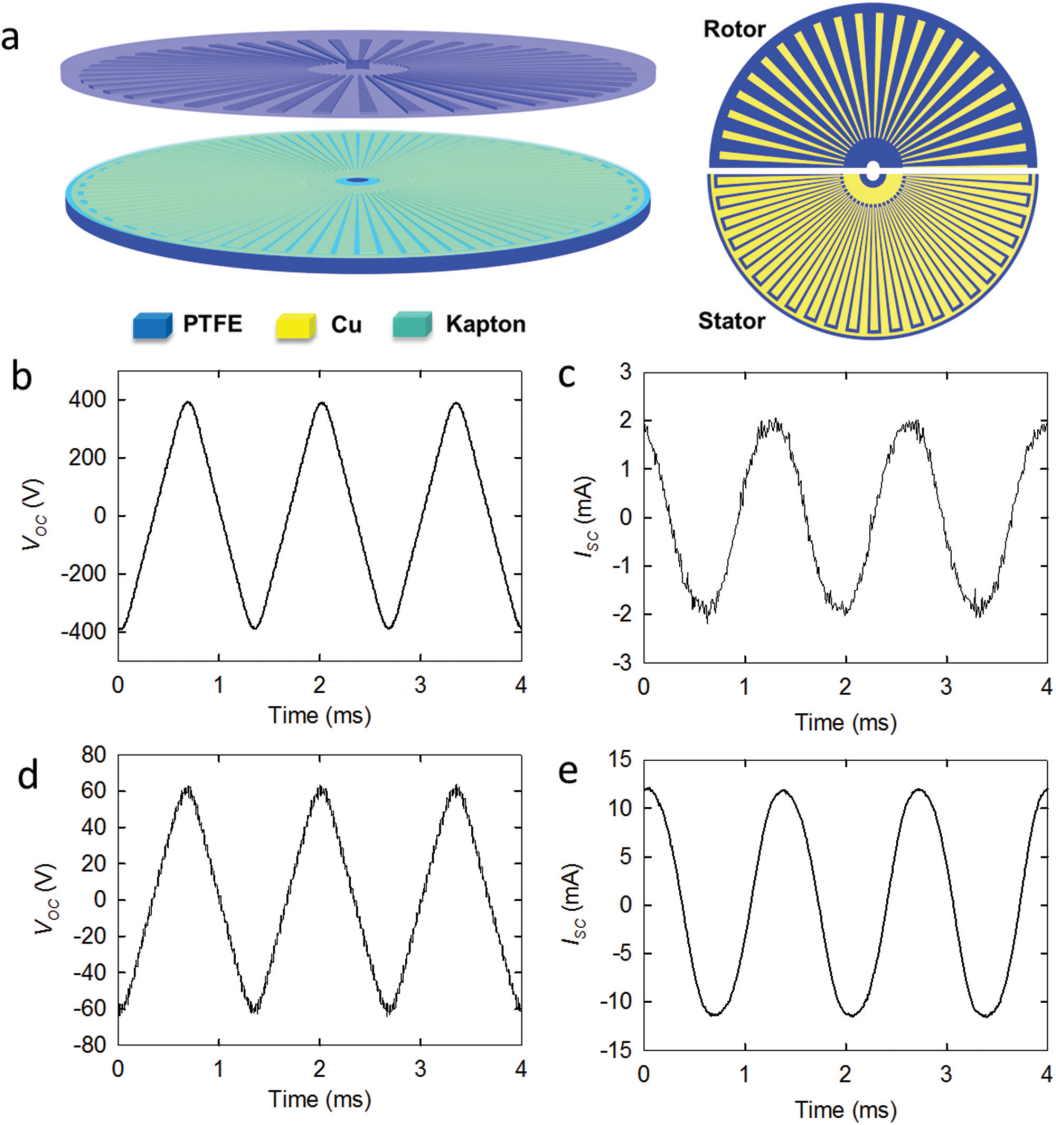
Output characteristics of triboelectric nanogenerator (TENG). a) Scheme of the TENG. Open‐circuit voltage (*V*_OC_) of TENG b) without and d) with a transformer (*n* = 36). Short‐circuit current (*I*_SC_) of TENG c) without and e) with the same transformer.

In order to address the challenges of the TENG outlined above, we chose to use a rotating TENG reported previously by our group and store its generated energy into lithium‐ion batteries.^12^ As shown in **Figure** **1**a, the TENG has a 2D planar structure, comprising of a bottom stator and a top rotor, both of which were fabricated based on the printed circuit board (PCB) technology. On the rotor board, there is a radial array of copper gratings, each of which has a length of 7.2 cm and central angle of 1°. On top of the stator, a radially interdigital structure of the two copper electrodes is covered by a Kapton thin film. Each “finger” of the underneath copper grating also has a central angle of about 1°. Once the rotor is brought into contact with the Kapton film and starts rotating, the Kapton film will be electrificated and electrons will flow alternately through an external circuit between the underneath two electrodes due to the electrostatic induction effect, generating an AC output. The open circuit voltage (*V*_OC_) of the TENG at 250 rpm rotating speed has a similar shape to the triangular wave with amplitude averaged at about 391 V, as shown in Figure 1b. The short‐circuit current (*I*_SC_) is a sinusoidal wave with amplitude of about 2 mA (see Figure 1c). The frequency of the AC output is about 750 Hz, corresponding well to the theoretical calculation by , where *f* is the output frequency, *N* is the rotating speed, and is the central angle of each grating.[[qv: 12b]] As the AC output can be tuned by a transformer, the amplitudes of *V*_OC_ and *I*_SC_ are transformed to ≈62 V (Figure 1d) and ≈11.6 mA (Figure 1e) by a transformer with coil ratio of *n* = 6.1, respectively, yielding a transfer efficiency of about ≈91(±3)%.

### 2.2 Battery Charging by the TENG

To demonstrate the feasibility of charging lithium‐ion batteries with the output current of the TENG, two half cells of LiFePO_4_ and Li_4_Ti_5_O_12_, typical electrode materials for cathode and anode, respectively, were assembled both with Li metal as the counter electrode. Figure S1 (Supporting Information) shows the rate performances of these two cells. When being charged/discharged galvanostatically at 0.5 C rate, the discharge capacity of LiFePO_4_ and Li_4_Ti_5_O_12_ half cell is 0.75 and 1.15 mAh, equivalent to specific capacity of 121.1 and 142.9 mAh g^−1^, respectively.

**Figure 2:**
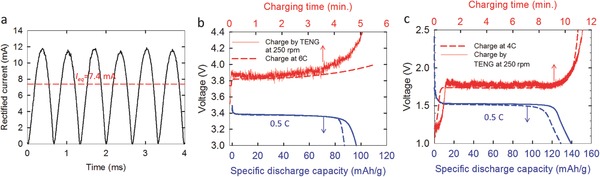
Li‐ion extraction of different electrode materials by the TENG. a) The short‐circuit current of the TENG used for battery charging, which is measured to be the same as the current when batteries are loaded. Voltage profiles of b) LiFePO_4_ and c) Li_4_Ti_5_O_12_ half cells charged by the TENG (solid lines) and constant current (dotted lines).

As the TENG has an AC output current, a bridge rectifier was used to reverse the negative portion to positive. After rectification, the equivalent DC current for generating the same quantity of charges can be calculated by: , which is independent of the frequency of the AC current. **Figure** **2**a shows the transformed (coil ratio *n* = 6.1) and rectified current of the TENG at 250 rpm, the *I*_eq_ of which is about 7.4 mA, as indicated by the horizontal dashed line. It should be noted that the current measured when the half cell was loaded is identical to the short‐circuit current, indicating that, even with the transformer, the internal impedance is still much higher than the battery cell. Detailed discussions on the impedance of batteries will be presented in later sections. By charging with the TENG for 5 min, the voltage of LiFePO_4_ half cell increased to 4.4 V, and the following discharge at 0.5 C constant current delivered a specific capacity of 96.4 mAh g^−1^ (solid line in Figure 2b), equivalent to 80% of that by 0.5 C rate galvanostatic charging for about 90 min (Figure S1a, Supporting Information), and higher than 87.2 mAh g^−1^ by 6 C rate galvanostatic charging for ≈5 min (dotted line in Figure 2b). A similar trend was observed in the Li_4_Ti_5_O_12_ half cell. An ≈11 min charging with the TENG later delivered a specific capacity of 139.1 mAh g^−1^ (solid line in Figure 2c), 97% of that by 0.5 C charging for 115 min (Figure S1b, Supporting Information), and higher than 128.9 mAh g^−1^ by 4 C rate charging for ≈11 min (dotted line in Figure 2c). This clearly indicates the reversible Li‐ion extraction or delithiation during the charging process, which can further be confirmed by X‐ray diffraction of the electrode materials before and after charging by the TENG, as shown in Figure S2 (Supporting Information).

One characteristic of the TENG charging, distinct from the galvanostatic charging, is the fluctuation of the voltage profiles, mainly due to the oscillation of the charging current. Because of the huge sampling rate (120 000 s^−1^) required for the accurate recording of the current, it is hard to record the current throughout the course of the TENG charging. Hence, the TENG charging capacity was roughly estimated by the product of *I*_eq_ and the charging time. Then, the columbic efficiencies of the TENG charging–0.5 C discharging process of LiFePO_4_ and Li_4_Ti_5_O_12_ half cells were estimated to be 92.5(±3.4)% and 90.1(±7.1)%, respectively, slightly smaller than 98%–100% of their galvanostatic counterparts. Chen et al. found that sinusoidal ripple current charging shortened charging time and improved columbic efficiency, comparing with charging by constant current–constant voltage.[[qv: 11a]] This discrepancy could be due to the possible error of our estimation arising from the misalignment of the rotor and stator.

**Figure 3:**
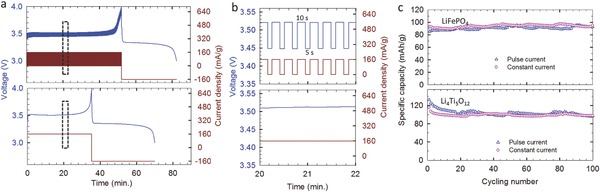
Charge of Li‐ion batteries with pulsed current. a,b) Comparison of voltage profiles of an LiFePO_4_ half cell (Li‐metal as counter electrode) charged by pulsed current (the upper plot) and constant current (the bottom plot) at 160 mA g^−1^. b) The enlarged view of the rectangular area in (a). c) Comparison of cycling performances of LiFePO_4_ (at 160 mA g^−1^) and Li_4_Ti_5_O_12_ (at 350 mA g^−1^), both with Li‐metal as counter electrode, charged by pulsed and constant current.

The pulsed AC output of the rotating TENG is different from that of many other low‐frequency TENGs, which typically have a period of rest time between adjacent current pulses. Therefore, a pulsed current was generated by a galvanostatic battery charger to mimic the charging process by the TENG. **Figure** **3**a,b compares the voltage profiles of an LiFePO_4_ half cell charged by a 1 C constant current (bottom plot in Figure 3a,b) and a pulsed current with duty ratio of 0.67 (top plot in Figure 3a,b). As shown in Figure 3b, the voltage oscillates with the pulsed charging current, similar to charging profiles in Figure 2b,c. Even though the charging time was elongated comparing with the constant current with the same amplitude, the discharge capacity and cycling performance showed negligible differences (Figure 3c), confirming the feasibility of battery charging by pulsed current. In fact, since 1970s, pulsed charging was developed as an advanced charging technique to even the ion distribution between the two electrodes, so as to lower the impedance and temperature increment during the charging process, and to improve the charging efficiency and cycling life.[[qv: 11b]]

### 2.3 Maximizing Charging Power

**Figure 4:**
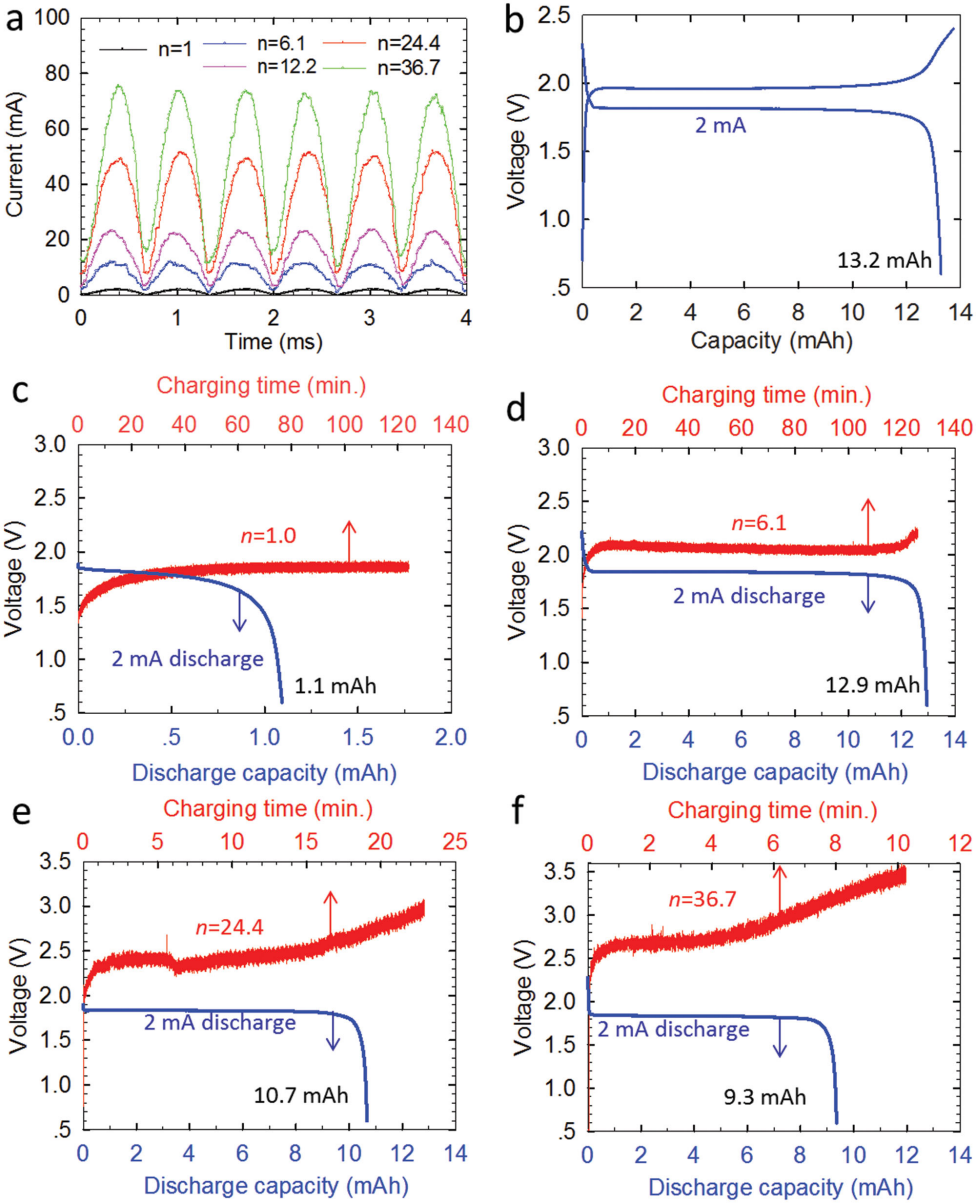
The effect of the transformer on the battery charging. a) Rectified currents of the TENG at the same rotating speed (250 rpm) but with different transformers (*n* = 1, 6, 12, 24, and 36). b) Voltage profiles of a home‐made LiFePO_4_–Li_4_Ti_5_O_12_ full cell charged and discharged by a 2 mA constant current. Voltage profiles of the cell charged by TENG at 250 rpm but with different transformers: b) *n* = 1.0, c) *n* = 6.1, d) *n* = 24.4, and e) *n* = 36.7.

Transformers with different coil ratios were utilized to tune the current of the TENG at 250 rpm for battery charging. As shown in **Table** **1** and Figure S4 (Supporting Information), when the coil ratio was increased to *n* = 24.4 and 36.7, the current amplitude rose to 48.4 and 73.5 mA, respectively. Corresponding voltage amplitude was then decreased to about 15 and 9 V, respectively. A battery pouch cell with LiFePO_4_ cathode and Li_4_Ti_5_O_12_ anode (LFP–LTO cell) was assembled. Comparing with rectified short‐circuit currents shown in **Figure** **4**a, we still observed no decrease of currents when the LFP–LTO cell was loaded for any of these transformers. When being galvanostatically cycled at 2 mA constant current, the LFP–LTO cell exhibited a charge voltage plateau of about 2 V, and delivered a 13.2 mAh discharge capacity (see Figure 4b).

**Table 1:** Charging LFP–LTO cell by the TENG with different transformers.

Transformer coil ratio *n*	Current amplitude [mA]	Voltage amplitude [V]	Charging time [min]	Discharging capacity [mAh]	Charging power [mW]	Power utilization efficiency [%]
1.0	2.0 ± 0.07	391.4 ± 1.8	125	1.1	2.4 ± 0.08	1.2 ± 0.04
6.1	11.6 ± 0.4	61.9 ± 0.4	125	12.9	16.1 ± 0.6	8.4 ± 0.3
24.4	48.4 ± 1.2	15.4 ± 0.1	23	10.7	76.9 ± 1.9	40.1 ± 1.0
36.7	73.5 ± 0.9	9.2 ± 0.4	10	9.3	139.0 ± 1.7	72.4 ± 0.9

After charging the LFP–LTO cell by the TENG, a discharge at 2 mA constant current was conducted to determine the stored capacity. Figure 4c–f shows the voltage profiles of the LFP–LTO cell charged by the TENG at 250 rpm with transformer coil ratio *n* = 1.0, 6.1, 24.4, and 36.7, respectively. The corresponding charging time and following discharge capacity are summarized in Table 1. When the coil ratio increased from *n* = 1.0 (i.e., without transforming) to 6.1, the discharge capacity by an ≈2 h charging dramatically boosted from 1.1 to 12.9 mAh, equivalent to 97.7% of the complete charge. Increasing the coil ratio further to 24.4 and 36.7 can shorten the charging time to about 23 and 10 min, but still deliver a discharge capacity of 10.7 and 9.3 mAh, respectively, equivalent to 81.1% and 70.5% of the complete charge, respectively. Clearly, the battery charging rate can thus be tuned by the coil ratio of the transformer.

The energy of the charging process with the TENG can be approximated by multiplying *I*_eq_ with the integration of the charging voltage profile, i.e., . Then, the power of the charging process can be determined by . As shown in Table 1, the charging power for *n* = 1.0, 6.1, 24.4, and 36.7 was 2.4, 16.1, 76.9, and 139.0 mW, respectively. The initial maximum power of the TENG was measured to be 192.0 mW when an external resistance of 150 kΩ was loaded. Without the transforming (*n* = 1.0), the power utilization efficiency is only about 1.2%, which however can be significantly improved to about 72.4% by a transformer with coil ratio of 36.7.

### 2.4 The Effect of the Rotating Speed

**Figure 5:**
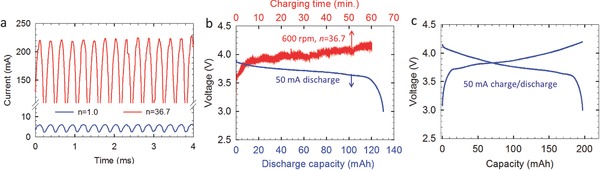
The battery charging by the TENG at 600 rpm. a) Rectified currents of the TENG without (*n* = 1) and with a transformer (*n* = 36). Voltage profiles of a commercial Li‐ion battery charged by the b) TENG and c) 50 mA constant current.

By raising the rotating speed, the amplitude and frequency of the output current of TENG will be both increased, but the amplitude of the voltage will not be changed.[[qv: 12b]] The output short‐circuit current of the TENG at 600 rpm showed an amplitude of 6 mA and a frequency of 1800 Hz (see Figure S5, Supporting Information). With transforming (*n* = 36.7), the current amplitude was increased to about 220 mA, as exhibited by the rectified current in **Figure** **5**a. A commercial Li‐ion battery (LiCoO_2_ cathode and graphite anode), possessing a capacity of 200 mAh when being galvanostatically cycled at 50 mA (Figure 5c), was utilized to store the energy generated by the TENG with the transformer. After charging for 1 h, the voltage of the cell increased to ≈4.2 V, and the subsequent discharging at 50 mA delivered a capacity of 130 mAh, equivalent to 65% of complete charge (see Figure 5b). The equivalent DC current *I*_eq_, independent of the frequency, can be estimated to be 140 mAh. Hence, the estimated columbic efficiency of this TENG charging–50 mA discharging process is about 92.7%. It should be noted that this capacity is enough to completely drive many small personal electronics, such as smart bracelets and watches. Meanwhile, considering the 2D planar design of the TENG and lightweight of the used materials, it is facile to stack many layers of TENG together to multiply the current and therefore the battery capacity. If assembled on the wheel of a bicycle or a car, the energy stored by the TENG is promising to power larger‐scale personal electronics, like smart phone and pad, which typically require battery capacity of ≈10^3^ mAh.

### 2.5 Impedance Match

**Figure 6:**
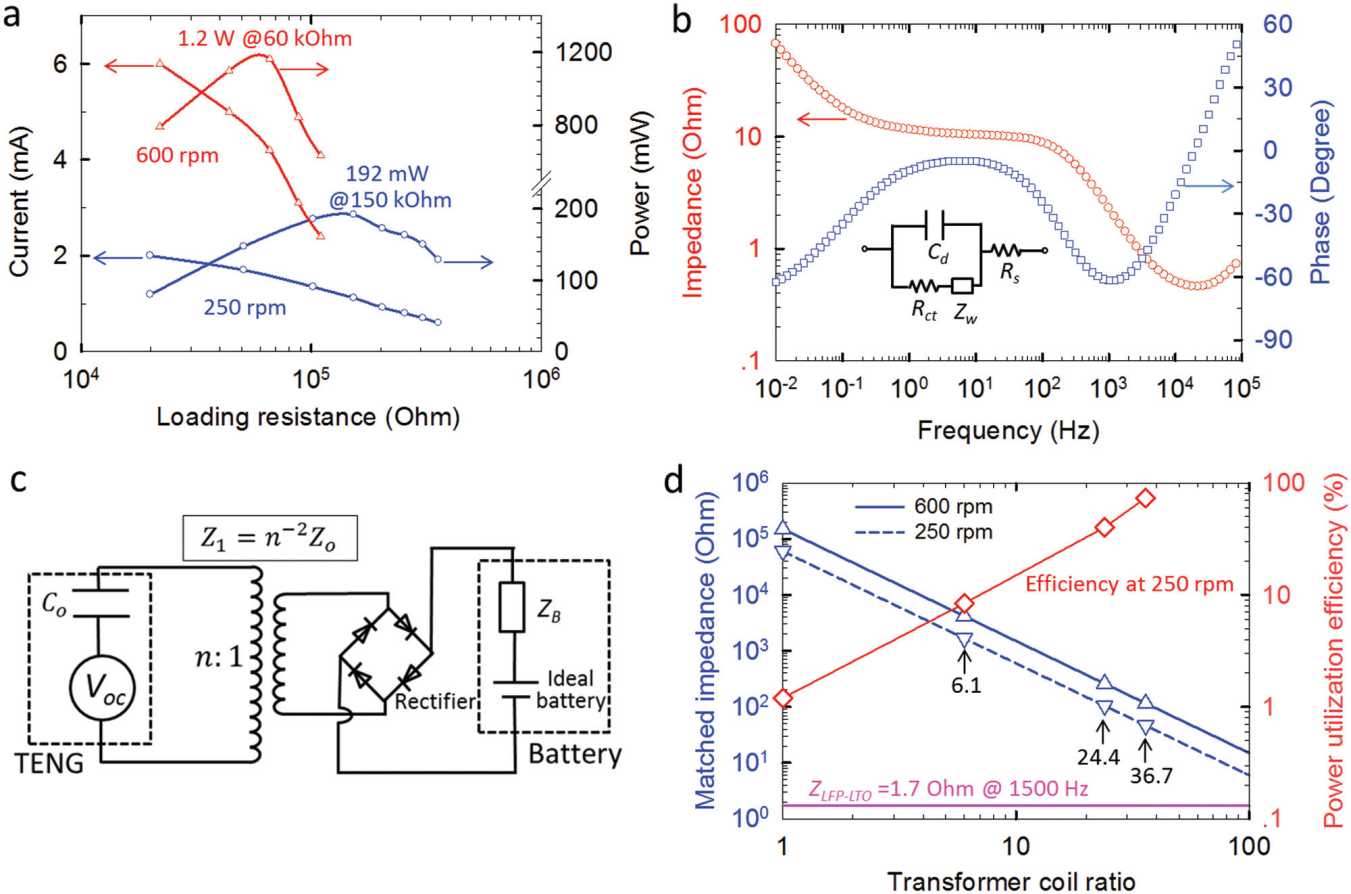
Improvement of the power utilization efficiency by impedance match. a) Variation of current and power of the TENG with the external resistance. b) Bode plot of impedances of an LFP–LTO full cell at discharged state. The inset in (b) is the equivalent circuit of the battery cell. c) The equivalent circuit of battery charging by the TENG with the aid of a transformer and a rectifier. d) The effect of the transformer coil ratio on matched impedances of the TENG, and the power utilization efficiency of the TENG at 250 rpm when charging an LFP–LTO full cell.

As outlined in the Introduction, the power utilization efficiency should be optimized when storing the energy of the TENG by a battery. Without transforming, the maximum power of 192 mW was achieved at matched external resistance of 150 kΩ if the rotating speed is 250 rpm; for rotating speed of 600 rpm, higher maximum power of 1.2 W was achieved at lower resistance of 60 kΩ (**Figure** **6**a). Nevertheless, the AC impedances of battery cells tested in this study were in the range of 0.1–300 Ω, as shown in Figure 6b and Figure S6 (Supporting Information). The typical equivalent impedance model *Z*_B_ of a battery contains an electrolyte resistance *R*_s_, a charging transfer resistance *R*_ct_, a Warburg impedance *Z*_w_, and a double layer capacitance *C*_d_ (see the inset in Figure 6b).[[qv: 11a]] *Z*_B_ is dependent on the AC frequency, exhibiting smaller impedance at higher frequency. Meanwhile, increasing the capacity of a battery leads to the decrease of the impedance, e.g., the 200 mAh battery showed impedances less than 1 Ω (Figure S6c, Supporting Information).

A transformer was found to be effective to bridge the gap between the battery impedance and matched impedance of the TENG. The equivalent circuit model of a TENG is analogous to a DC voltage source *V*_oc_ in series with a capacitor *C*_o_ ;^10^ while a battery is analogous to an ideal battery in series with an impedance *Z*_B_ (Figure 6c).[[qv: 11a]] By a transformer with coil ratio of *n*, the impedance will be decreased to . Hence, impedance match can be achieved by appropriate design of the transformer, i.e., .

Our above result of charging LFP–LTO cell with the TENG at 250 rpm is just an example on how optimized impedance match can affect the power utilization efficiency. The current after rectification (at 250 rpm) has a frequency of 1500 Hz. The corresponding impedance of the LFP–LTO cell was determined to be 1.7 Ω (Figure 6b), ≈5 orders of magnitude lower than matched impedance of the initial TENG. This huge discrepancy resulted in the extremely low charging power of 2.4 mW and the power utilization efficiency of 1.2% (Table 1 and Figure 6d). With a transformer (*n* = 36.7), the matched impedance decreased to about 110 Ω, and the charging power was improved to 139.0 mW, equivalent to the utilization efficiency of 72.4% (Table 1 and Figure 6d).

## 3 Conclusions

In summary, we demonstrated the feasibility of high‐efficient charging lithium‐ion batteries with pulsed output currents of the TENG. Fast and reversible delithiation of LiFePO_4_ and Li_4_Ti_5_O_12_ were achieved by a rotating TENG with AC current output. The estimated columbic efficiency of the TENG charging and galvanostatic discharging was higher than 90% as well. Pulsed current generated by a galvanostatic battery charger also showed negligible difference to battery galvanostatic charging, further indicating the feasibility of battery charging with pulsed output of the TENG. An LFP–LTO full cell (≈13.2 mAh) was charged by the TENG at 250 rpm with different transformers, i.e., coil ratio *n* = 1.0, 6.1, 24.4, and 36.7. A larger coil ratio yielded a higher charging current, and therefore shortened the charging time and improved the charging power. When *n* = 36.7, a 10 min charging was equivalent to 70.4% full charge. The charging current can also be tuned by the rotating speed. At 600 rpm and transformer ratio of 36.7, the current amplitude can be 220 mA, yielding a capacity of 130 mAh for a 1 h charging of a commercial LiCoO_2_–C battery. Finally, the improvement of power utilization efficiency by appropriate design of transformer was discussed. When the matched impedance of the TENG was lowered close to that of the battery, a higher charging power and power utilization efficiency will be achieved. In this study, an efficiency of 72.4% was achieved when charging the LFP–LTO cell with the transformer coil ratio of 36.7.

## 4 Experimental Section

*Fabrication of the TENG*: The TENG with a configuration shown in Figure 1a was fabricated based on PCB technology.

*Assembly of Battery Cells*: Chemicals related to batteries were all purchased from MTI, Inc. Electrodes of LiFePO_4_ and Li_4_Ti_5_O_12_ were made by coating the active slurry onto Al and Cu foil with a doctor blade, respectively. The slurry was a mixture of the active materials, carbon black as conducting additive, and Poly(vinylidene fluoride) (PVDF) as polymer binder (at weight ratio of 7:2:1). For assembly of the half cell, an LiFePO_4_ (or Li_4_Ti_5_O_12_) electrode and an Li metal disc were stacked together and sealed in a 2023 coin cell with a Celgard 2325 separator membrane in between; for the full cell, electrodes of LiFePO_4_ and Li_4_Ti_5_O_12_, as cathode and anode respectively, were sealed into an Al pouch cell. Electrolyte of 1 m LiPF_6_ in ethylene carbonate (EC) and dimethyl carbonate (DMC) (1:1 by vol.) was used for all these cells. The battery assembly was conducted in an Ar‐filled glove box with O_2_ and H_2_O content both less than 0.1 ppm.

*Characterization*: The short‐circuit current and open‐circuit voltage of the TENG were measured by an electrometer (Keithley 6517B) and an oscillometer (Lecory HDO6104), respectively. The galvanostatic charging/discharging of battery cells was conducted by a commercial battery cycler (LAND CT2001A). AC impedances (from 100 kHz to 0.01 Hz, and voltage amplitude of 5 mV ) of batteries were measured by an electrochemical work station (CHI 660E) at discharged state. To charge the battery cell, transformers with different coil ratio and a bridge rectifier were applied. When charging batteries with the TENG, the cell voltage was recorded by the Keithley 6517B. The galvanostatic discharging was subsequently carried out to determine the charged capacity.

## Supporting information

As a service to our authors and readers, this journal provides supporting information supplied by the authors. Such materials are peer reviewed and may be re‐organized for online delivery, but are not copy‐edited or typeset. Technical support issues arising from supporting information (other than missing files) should be addressed to the authors.

Supplementary

## References

[1] a) F. J. DiSalvo , Science 1999, 285, 703; 10426986 10.1126/science.285.5428.703

[2] a) Z. L. Wang , J. Song , Science 2006, 312, 242; 16614215 10.1126/science.1124005

[3] a) Y.‐H. Lee , J.‐S. Kim , J. Noh , I. Lee , H. J. Kim , S. Choi , J. Seo , S. Jeon , T.‐S. Kim , J.‐Y. Lee , J. W. Choi , Nano Lett. 2013, 13, 5753; 24164580 10.1021/nl403860k

[4] a) C. R. Saha , T. O'Donnell , N. Wang , P. McCloskey , Sens. Actuators, A 2008, 147, 248;

[5] a) C. Dong‐Hoon , H. Chang‐Hoon , K. Hyun‐Don , Y. Jun‐Bo , Smart Mater. Struct. 2011, 20, 125012;

[6] a) X. Wang , Nano Energy 2012, 1, 13;

[7] a) F.‐R. Fan , Z.‐Q. Tian , Z. L. Wang , Nano Energy 2012, 1, 328;

[8] a) G. Zhu , B. Peng , J. Chen , Q. Jing , Z. L. Wang , Nano Energy 2015, 14, 126;

[9] a) S. Niu , Z. L. Wang , Nano Energy 2015, 14, 161;

[10] S. Niu , Y. Liu , Y. S. Zhou , S. Wang , L. Lin , Z. L. Wang , IEEE Trans. Electron Devices 2015, 62, 641.

[11] a) C. Liang‐Rui , W. Shing‐Lih , S. Deng‐Tswen , C. Tsair‐Rong , IEEE Trans. Ind. Electron. 2013, 60, 88;

[12] a) G. Zhu , J. Chen , T. Zhang , Q. Jing , Z. L. Wang , Nat. Commun. 2014, 5, 3426; 10.1038/ncomms4426 24594501

