# Supplementary material for: Efficient Charging of Li‐Ion Batteries with Pulsed Output Current of Triboelectric Nanogenerators
Source: Adv Sci (Weinh). 2015 Sep 25;3(1):1500255. doi: 10.1002/advs.201500255 (PMC5054865; doi:10.1002/advs.201500255)
Supplement: Supplementary file 1 — Supplementary [file ADVS-3-0a-s001.pdf]

Copyright WILEY-VCH Verlag GmbH & Co. KGaA, 69469 Weinheim, Germany, 2015.

ADVANCED SCIENCE
Open Access

Supporting Information

for *Adv. Sci.*, DOI: 10.1002/advs. **201500255**

Efficient Charging of Li-Ion Batteries with Pulsed Output Current of Triboelectric Nanogenerators

*Xiong Pu , Mengmeng Liu , Linxuan Li , Chi Zhang , Yaokun Pang , Chunyan Jiang , Lihua Shao , Weiguo Hu , * and Zhong Lin Wang **

Copyright WILEY-VCH Verlag GmbH & Co. KGaA, 69469 Weinheim, Germany, 2013.

Supporting Information

Efficient Charging of Li-ion Batteries with Pulsed Output Current of Triboelectric Nanogenerators

Xiong Pu, Mengmeng Liu, Linxuan Li, Chi Zhang, Yaokun Pang, Chunyan Jiang, Lihua Shao, Weiguo Hu, Zhong Lin Wang**

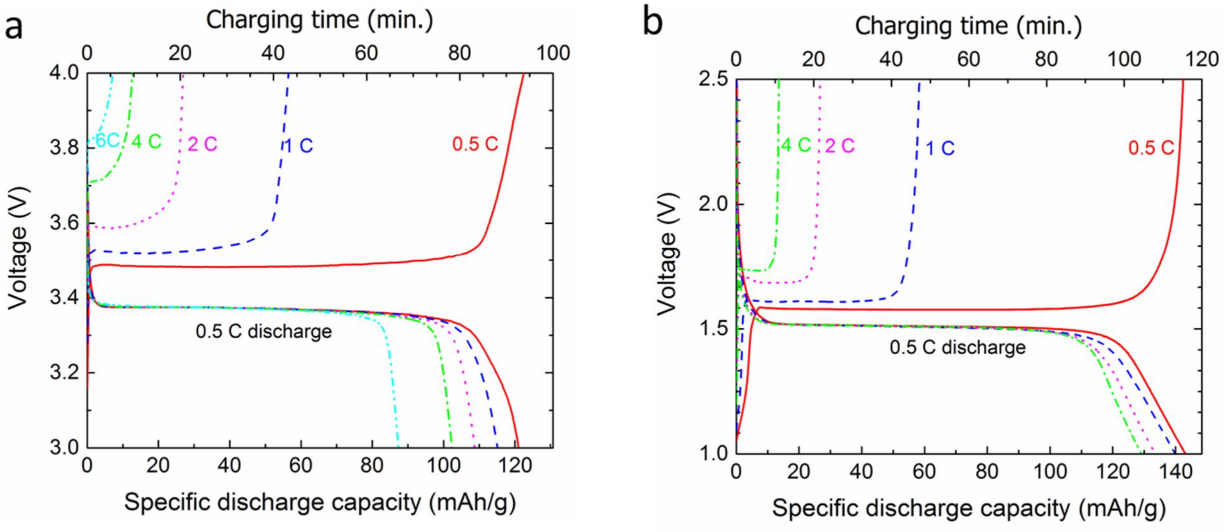

Figure S1. Voltage profiles of $LiFePO_4$ (a) and $Li_4Ti_5O_{12}$ (b) half cells charged at different C rates and discharged at 0.5 C rate.

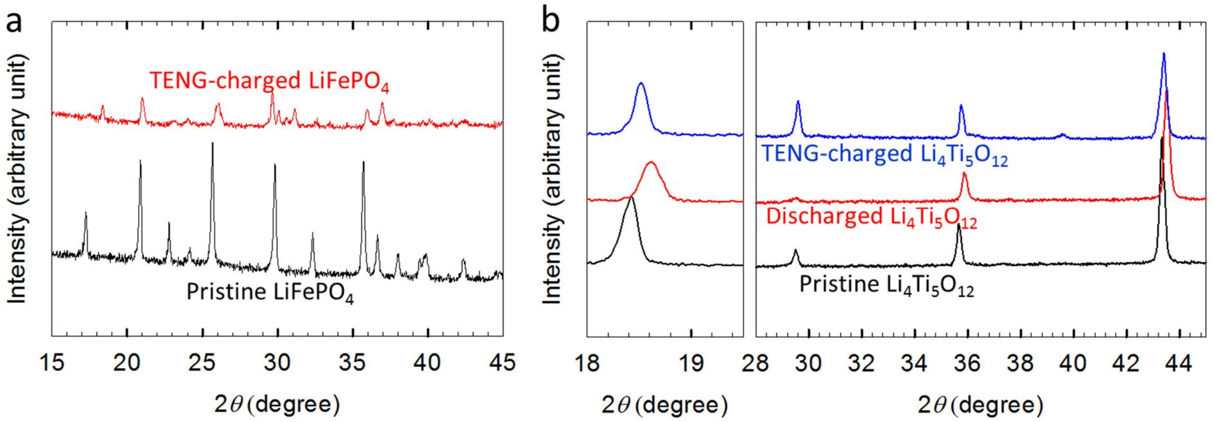

Figure S2. X-ray diffraction patterns of $LiFePO_4$ (a) and $Li_4Ti_5O_{12}$ (b) before and after the charge by TENG.

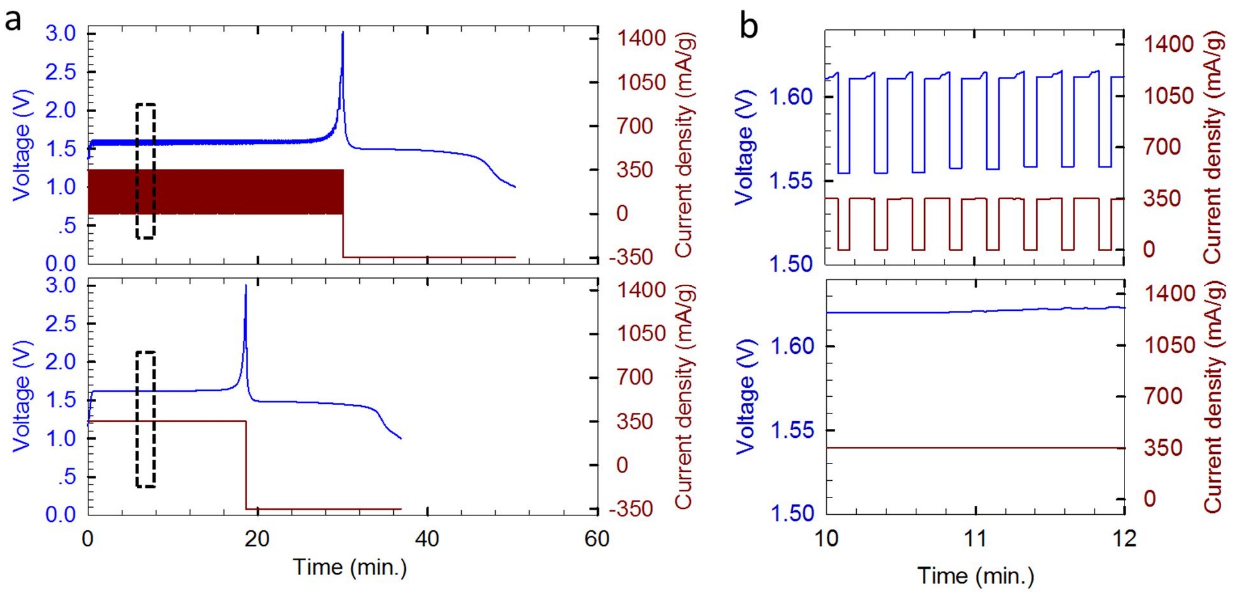

Figure S3. Voltage profiles of $Li_4Ti_5O_{12}$ half cell charged by pulsed current (the upper plot) and constant current (the bottom plot) at 350 mA/g. (b) is the enlarged view of the rectangular area in (a).

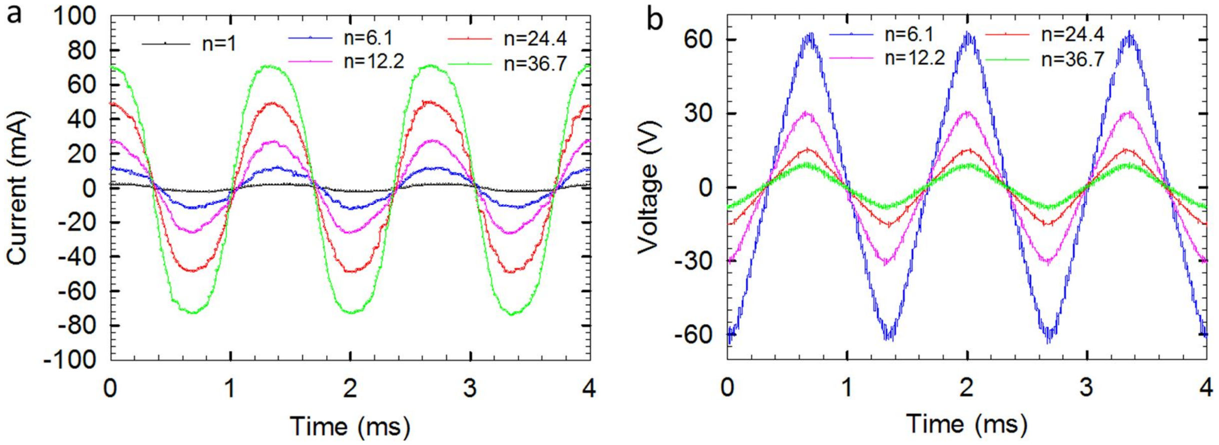

Figure S4. Short-circuit current (a) and open-circuit voltage (b) of the TENG at 250 rpm with different transformer coil ratio *n*.

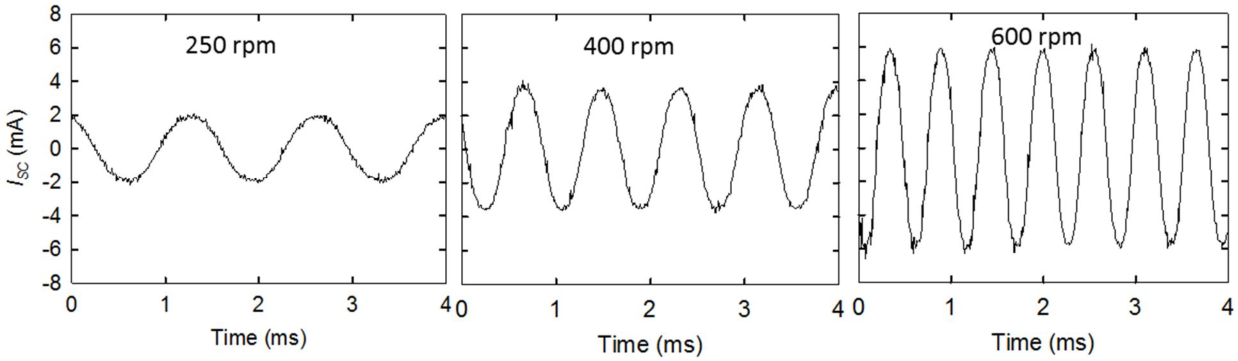

Figure S5. Short-circuit current of the TENG at different rotation speed without transforming, i.e 250 rpm, 400 rpm, 600 rpm.

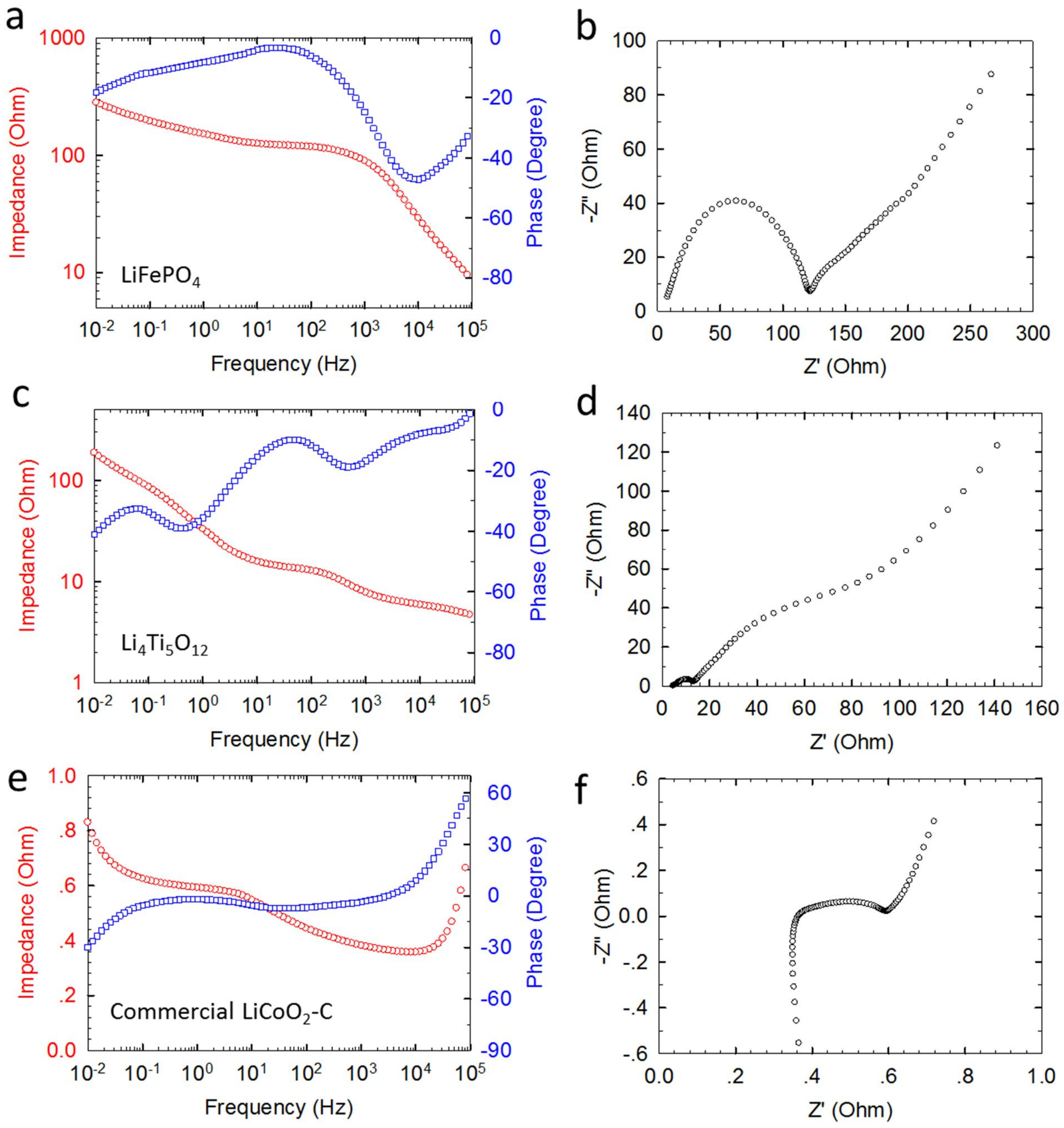

Figure S6. Bode plot (a, c and e) and Nyquist plot (b, d and f) of the impedances of $LiFePO_4$ (a and b) half cell, $Li_4Ti_5O_{12}$ (c and d) half cell, and a commercial $LiCoO_2$-C full cell (~200 mAh) at discharged state.
